# Scheduling Strategy Design Framework for Cyber–Physical System with Non-Negligible Propagation Delay

**DOI:** 10.3390/e23060714

**Published:** 2021-06-04

**Authors:** Zuoyu An, Shaohua Wu, Tiange Liu, Jian Jiao, Qinyu Zhang

**Affiliations:** Communication Engineering Research Centre, Harbin Institute of Technology (Shenzhen), Shenzhen 518055, China; 19s052043@stu.hit.edu.cn (Z.A.); 19s152063@stu.hit.edu.cn (T.L.); jiaojian@hit.edu.cn (J.J.); zqy@hit.edu.cn (Q.Z.)

**Keywords:** cyber–physical system, wireless networked control system, remote control, communication control co-design, age of information

## Abstract

Cyber–physical systems (CPS) have been widely employed as wireless control networks. There is a special type of CPS which is developed from the wireless networked control systems (WNCS). They usually include two communication links: Uplink transmission and downlink transmission. Those two links form a closed-loop. When such CPS are deployed for time-sensitive applications such as remote control, the uplink and downlink propagation delay are non-negligible. However, existing studies on CPS/WNCS usually ignore the propagation delay of the uplink and downlink channels. In order to achieve the best balance between uplink and downlink transmissions under such circumstances, we propose a heuristic framework to obtain the optimal scheduling strategy that can minimize the long-term average control cost. We model the optimization problem as a Markov decision process (MDP), and then give the sufficient conditions for the existence of the optimal scheduling strategy. We propose the semi-predictive framework to eliminate the impact of the coupling characteristic between the uplink and downlink data packets. Then we obtain the lookup table-based optimal offline strategy and the neural network-based suboptimal online strategy. Numerical simulation shows that the scheduling strategies obtained by this framework can bring significant performance improvements over the existing strategies.

## 1. Introduction

In the recent past, applications of the wireless control networks have become more and more extensive, such as drone formations, autonomous vehicles, automatic factories, etc. Some of those scenarios implicate new requirements for remote control technology, which is a sub-topic of communication control co-design. Remote control technology originates from wireless control systems with long propagation delay such as far-sea monitoring and high-efficiency satellite IoT. The main cause of long propagation delay is the large-scale geographic distance. This feature makes it extremely challenging to design CPS under this scenario. In order to meet the need of remote control with propagation delay, that is, to maintain stable closed-loop control and reduce control costs, we propose a new framework to design uplink and downlink scheduling strategies.

**Figure 1:**
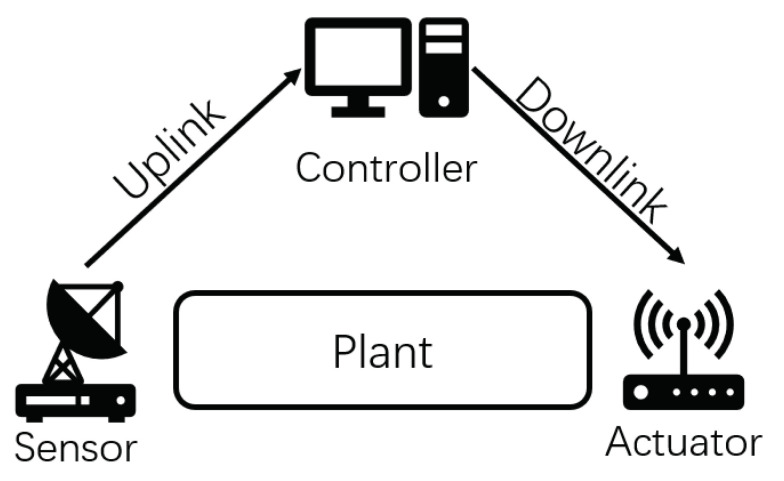
Cyber–physical system deployed under the single closed-loop control scenario.

As show in Figure 1, a typical CPS deployed under the single closed-loop control scenario contains a control system and a communication system. In the rest of this article, we use single-loop CPS to refer to this specific type of CPS. The communication process of a typical single-loop CPS can be divided into two parts: Uplink sensor transmission and downlink controller transmission. The uplink transmission is initiated by the sensor and sends the state update packet from the plant to the controller. The controller first uses this data to obtain a more accurate estimate of the factory status. Then the downlink transmission is initiated to send command information from the controller to the actuator located at the factory. The actuator acts on the factory to maintain the factory’s stability. Taking into account the characteristics of a control system, the command can only be generated with an accurate estimation, which means the downlink transmission must occur after a successful uplink transmission. Because of this fixed timing relationship, CPS has to work in half-duplex in most cases: namely, only one of the uplink sensor transmission and the downlink controller transmission can be activated to send a data packet in the same time slot. That means there is a problem of how to design a scheduling strategy between those two transmissions. Note that the uplink and downlink channels here are not just a single wireless channel, but a simplified modeling of a fixed routing link with multiple relays. This scenario is for some special remote control systems that use satellites as relays. Therefore, the propagation delay in our paper is essentially a collection of various delays contained in the entire relay link, including processing delay, transmission delay, propagation delay, etc. This unified modeling is used because the link characteristics of a fixed routing multi-relay link can be described by an equivalent link with a specific code error rate and propagation delay.

There are many related works about WNCS and CPS [1,2,3,4]. Focusing on the conflict of the accuracy requirements of control systems and the limited quantization level [5], proposed the application of dynamic quantization technology in the communication control co-design. Some works designed CPS with the limitation of wireless coding process, such as code length allocation [6,7], code length design [8,9] and adaptive code length adjustment [10]. Considering the fading characteristics of transmission channels, studies of adaptive transmit power adjustment technology by predicting the fast or slow fading of transmission channels are proposed in [11,12]. Some of the above studies include the idea of designing CPS for time-sensitive applications. Nowadays, the most widely used measure of timeliness is Age of Information (AoI) [13], which is defined as the time elapsed since a certain data packet was generated:

(1) $$\Delta \left(t\right)\;=\;t-t^{\prime }$$

where *t* represents the current time, $t^{\prime }$ represents the time when the packet was generated. It used to be very difficult to express the control performance measurement, that is, the system state mean square error (MSE) [14] when the control system and the communication system are combined. The proposal of AoI changed this situation. For example, the system state MSE of a linear time invariant system (LTI) can be simply expressed as a function of AoI. This improvement greatly reduces the difficulty of describing the overall system performance in the communication control co-design scenario [15,16].

Based on AoI, many related studies have been derived, such as the application of the HARQ mechanism for single-loop CPS to improve the overall timeliness [17,18], and the scheduling strategy aiming to minimize the long-term average MSE for single-loop CPS without transmission delay [19]. Some studies about the multi-loop scheduling strategy design aiming at optimizing timeliness have also been proposed. Reference [20] focuses on the design of the data inter-arrival rate and code length allocation strategy. References [21,22] proposed the uplink scheduling strategy of multi-loop WNCS under the ideal assumption of downlink transmission. Furthermore, the authors of [23,24] discuss the application of data packet transmission result prediction technology in WNCS design.

The scenarios studied above concern mainly short-distance Industrial Internet of Things (IIoT), so the impact of uplink and downlink propagation delay on the closed-loop control performance of a CPS is generally ignored. Besides, the above studies only consider one of the two code error rates of the uplink and the downlink transmission. Under the remote control scenario, the code error rates and propagation delay of both links are not only non-negligible, but also have a huge impact on the overall performance of the single-loop CPS. Some works have studied the design of WNCS optimal control strategy under time-delay scenarios [25,26,27]. However, they do not consider the impact of the code error rate and the scheduling strategy which are issues that cannot be ignored in the design of communication systems in the field of communication engineering. To this end, we propose a new framework to obtain the optimal scheduling strategy while considering both the code error rates and propagation delay. This strategy can minimize the long-term average control cost.

Firstly, we model the single-loop CPS as an MDP problem and give the sufficient conditions for the stability of CPS. Secondly, we propose a heuristic semi-predictive framework to eliminate the impact of the coupling characteristic between the uplink and downlink data packets. Finally, we obtain the lookup table-based optimal offline strategy and the neural network-based suboptimal online strategy for the single-loop CPS. The whole process can be expanded according to actual deployment requirements with any fixed propagation delay as long as the sufficient condition is satisfied.

The rest of this paper is organized as follows: In Section 2, we provide the system model and formulate the optimization problem. In Section 3, we introduce the semi-predictive framework and transform the optimization problem into an MDP problem. In Section 4, we obtain the optimal offline strategy and the suboptimal online strategy. In Section 5, we show the numerical simulation results. We conclude this work in Section 6.

## 2. System Model

### 2.1. The Plant of the Single-Loop CPS

First, we model the plant in the single-loop CPS as a discrete-time LTI system:

(2) $$X_{k+1}=AX_{k}+BU_{k}+Z_{k},\forall k$$

where *k* represents the *k*-th time slot, $X_{k}\in R$ represents the state of the plant at time slot *k*, $U_{k}\in R$ represents the executed control command, $Z_{k}\in R$ represents the normally distributed plant noise whose mean and variance are $\bar{z}$ and *R*, respectively. A∈R represents the state transition coefficient, B∈R represents the command control coefficient. We assume that the plant state remains unchanged within a single time slot. The goal of CPS is to maintain *X* around 0.

### 2.2. The Communication Process of the Single-Loop CPS

In the previous subsection, we explained that the entire single-loop CPS works in the half-duplex mode. Now we will explain the communication process of the single-loop CPS. The entire system adopts a centralized scheduling scheme because this scheme is more suitable for single-loop CPS. Under this scheme, the scheduling decision of uplink and downlink transmission is completely determined by the remote controller. We use $a_{k}$ to represent the scheduling decision made by the controller in the time slot *k*. If the controller schedules uplink transmission in the slot *k*, $a_{k}\;=\;1$. If the controller schedules downlink transmission in the slot *k*, $a_{k}\;=\;2$. We assume that the code error rate of the uplink and downlink transmission channels are $p_{s},p_{c}\in \left(0,1\right)$, respectively. Both code error rates are constant which means the uplink and downlink transmission fails with probability $\left(p_{s},p_{c}\right)$ in any time slot, respectively. Then we use $\delta_{k}$ to represent the transmission result of the packet sent in the time slot *k*. No matter which transmission is scheduled, if it succeeds, then $\delta_{k}\;=\;1$. Otherwise, $\delta_{k}\;=\;0$. Since the processing procedures of most actual CPS are digital, the packets that have experienced a certain delay will start to be processed in the next processing cycle after it is received; we model the propagation delay of the uplink and downlink channel integer time slots $d_{\mathrm{up}},d_{\mathrm{down}}\in R$, respectively. To simplify the analysis, we assume that the transmission of scheduling instructions and feedback information is ideal.

In addition to the variables described above, we define the following two parameters to describe the status of each part in a single-loop CPS:

(1) State Estimation Age $\tau_{k}$: This is defined as the age of the latest valid uplink state update packet successfully received by the controller at the end of the time slot *k*. $\tau_{k}$ reflects the accuracy of the estimation maintained by the remote controller. Because of the uplink propagation delay, the minimum value of state estimation age is $d_{\mathrm{up}}$. When the specific time slot is not considered, it is abbreviated as τ. Its update rule is as follows:

(3) $$\tau_{k+1}=\left\{\begin{array}{cc} d_{\mathrm{up}} & \mathrm{if}\;\;\;\;\left(a_{j}=1\;\right)\&(\delta_{j}=1)\\ \tau_{k}+1 & \mathrm{otherwise} \end{array}\right.$$

where $j=k-d_{\mathrm{up}}+1$.

(2) State Control Age $\varphi_{k}$: This is defined as the age of the uplink packet used to generate the latest successfully received downlink packet by the actuator at the end of the time slot *k*. This parameter represents the total time it takes for the entire CPS to complete a closed-loop control process. It reflects the degree of divergence of the plant’s state. Because of the uplink and downlink propagation delay, the minimum value of the state control age is $d_{\mathrm{up}}+d_{\mathrm{down}}$. When the specific time slot is not considered, it is abbreviated as φ. Its update rule is as follows:

(4) $$\varphi_{k+1}=\left\{\begin{array}{cc} \tau_{q}+d_{\mathrm{down}} & \mathrm{if}\;\;\;\;\;\left(a_{q}=2\right)\&(\delta_{q}=1)\\ \varphi_{k}+1 & \mathrm{otherwise} \end{array}\right.$$

where $q=k-d_{\mathrm{down}}+1$. The abbreviations *j* and *q* will be used in the rest of this paper. Note that we set the initial values of $\tau_{0}$ and $\varphi_{0}$ to be 2. These values can be arbitrarily selected within a reasonable range. This is because the long-term average cost we focus on is not affected by those initial values.

### 2.3. The Control Process of the Single-Loop CPS

In this subsection, we will explain the control process of the single-loop CPS in detail, which is mainly completed by the remote controller and the actuator. The task of the remote controller can be divided into three parts: Maintaining state estimation, generating control commands, and scheduling uplink and downlink transmissions, while the actuator has only one task: Executing the received control commands.

(1) Maintaining State Estimation: We assume that the sensor can sample the state of the plant without distortion. The uplink transmission cannot be scheduled in every time slot. What is more, the scheduled transmission can fail because of the code error occurring during its propagation process. So the remote controller cannot receive a new state update packet in every time slot. Under these circumstances, the remote controller has to update the estimation ${\tilde{X}}_{k}$ of the plant state $X_{k}$ through the following process:

(5) $${\tilde{X}}_{k\;+\;1}=\left\{\begin{array}{cc} g^{d_{\mathrm{up}}}\left(X_{j},k\right) & \mathrm{if}\;\;\;\;\left(a_{j}=1\;\right)\&(\delta_{j}=1)\\ A{\tilde{X}}_{k}+BU_{k} & \mathrm{otherwise} \end{array}\right.$$

where $g\left(X,k\right)=AX+BU_{k}$, $g^{n}\left(X,k\right)=g\left(g^{n-1}\left(X,k-1\right),k\right)\;\forall n>1$, and $g^{1}\left(X,k\right)=g\left(X,k\right)$. In this scenario, this estimation method has been proven to be optimal [28]. When a certain uplink transmission is successful, the remote controller can use the plant state $X_{k-d_{\mathrm{up}}+1}$, which is the exact value for $d_{\mathrm{up}}-1$ time slots before, to obtain the state estimation ${\tilde{X}}_{k\;+\;1}$ of the next time slot. When the current time slot has no successful uplink transmission, the controller can only update ${\tilde{X}}_{k\;+\;1}$ with ${\tilde{X}}_{k}$. According to this process, we can derive the state estimation MSE of the remote controller as ${\tilde{Q}}_{k}$:

(6) $${\tilde{Q}}_{k}\;=\;E\left[{\left({\tilde{X}}_{k}-X_{k}\right)}^{2}\right]$$

Note that the state estimation error of the remote controller is entirely caused by the noise $Z_{k}$. By using the state estimation age $\tau_{k}$, we can rewrite the state estimation MSE as a recursive function of the noise variance *R*:

(7) $${\tilde{Q}}_{k+1}\;=\;\left\{\begin{array}{cc} f(d_{\mathrm{up}}) & \mathrm{if}\;\;\;\;\left(a_{j}=1\;\right)\&(\delta_{j}=1)\\ f(\tau_{k}+1) & \mathrm{otherwise} \end{array}\right.\;$$

where $f\left(x\right)=\sum_{i=1}^{x}{\left(A^{2}\right)}^{i\;-\;1}R$. Equation (6) uses the definition of AoI to derive the MSE of the estimation. This representation greatly reduces the difficulty of calculation. In the following part, we will use the same idea to derive the single-loop CPS control performance metrics.

(2) Control Command Generation and Execution: In each time slot, while the remote controller maintains the state estimation, it also uses the estimation to generate a control command ${\tilde{U}}_{k}$:

(8) $${\tilde{U}}_{k}\;=\;K{\tilde{X}}_{k}$$

where *K* is the command generation coefficient. The goal of this control process is to maintain the state around 0. Since the downlink transmission has a propagation delay of $d_{\mathrm{down}}$ time slots, we must ensure $BK=-A^{d_{\mathrm{down}}}$. To simplify the analysis, we set $B=-A^{d_{\mathrm{down}}}$, K=1. Due to the code error rate and scheduling decisions, not every control command $\tilde{U}$ can be received by the actuator. Only those scheduled and successfully transmitted can be used by the actuator. Therefore, the control command executed by the actuator is $U_{k+1}$:

(9) $$U_{k+1}=\left\{\begin{array}{cc} {\tilde{U}}_{q} & \mathrm{if}\;\;\left(a_{q}=2\;\right)\&(\delta_{q}=1)\\ 0 & \mathrm{otherwise} \end{array}\right.$$

where $q=k-d_{\mathrm{down}}+1$. This control method shown by (8) and (9) is called single-step control, which is a common form in the field of classic cybernetics. Using this method, when a control command is successfully delivered to the actuator, the actual state value will return to a value as close to 0 as possible at one time. Such a process can maximize the effect of a single instruction.

(3) Single-Loop CPS Control Performance Metrics: Consistent with the estimation performance metrics, the control performance metrics is defined as the state MSE of the plant $Q_{k}$:

(10) $$Q_{k}\;=\;E\left[X_{k}^{2}\right]$$

Similar to ${\tilde{Q}}_{k}$, we can rewrite $Q_{k}$ as a function of noise variance *R* and state control age φ:

(11) $$Q_{k+1}\;=\;\left\{\begin{array}{cc} f(\tau_{q}+d_{\mathrm{down}}) & \mathrm{if}\;\;\;\;\;\left(a_{q}=2\right)\&\left(\delta_{q}=1\right)\\ f(\varphi_{k}+1) & \mathrm{otherwise} \end{array}\right.\;$$

According to the control cost given by Equation (11), we can obtain the long-term average control cost, that is, the long-term average plant state MSE:

(12) $$J=\lim_{K\to \infty }\frac{1}{K}\sum_{k=0}^{K}Q_{k}$$

Equation (12) reflects the state deviation in the field of classic cybernetics which is the core cost metrics we care about. Please note that this parameter used to be very difficult to quantify without the introduction of AoI. Under certain conditions, the limit contained in Equation (12) may not exist, and the problem is unsolvable. In order to prevent such situations, the sufficient condition for the stability of WNCS with propagation delay will be given later, namely Equation (19). In this paper, the scheduling strategy will be designed on the premise that Equation (19) is satisfied.

(3) Uplink and Downlink Scheduling Process: In the previous subsection, we introduced the control performance measurement of a single-loop CPS. Now we will describe the scheduling process in detail. It has been explained that a single-loop CPS has two communication scenarios—the uplink transmission and the downlink transmission—and we can only choose one of them in each time slot under half-duplex mode. According to the previous definition, the scheduling decision of time slot *k* is recorded as $a_{k}$. The set of scheduling decisions of all time slots is called a scheduling strategy:

(13) $$\pi ≜(a_{1},a_{2},\ldots ,a_{k},\ldots )\in \Pi$$

where Π represents the set of all scheduling strategies. Different scheduling strategies can significantly affect the control performance of a single-loop CPS. Every scheduling strategy π has its corresponding long-term average control cost $J_{\pi }$. Among all scheduling strategies, there is an optimal strategy $\pi^{*}\in \Pi$, which satisfies:

(14) $$J_{\pi^{*}}⩽J_{\pi },\;\forall \pi \in \Pi$$

Therefore, we can construct the following optimization problem. The goal of this problem is to minimize the long-term average plant state MSE to obtain the optimal scheduling strategy while taking transmission propagation delay and code error rates of two wireless channels into account, namely

(15) $$\min_{\pi }\lim_{K\to \infty }\frac{1}{K}\sum_{k=0}^{K}Q_{k}$$

## 3. Semi-Predictive Framework and MDP Modeling

In this section, we will introduce the coupling characteristic between the uplink and downlink data packets which is caused by their propagation delay. In the following paper, we will use the coupling characteristic to refer to the coupling characteristic between the uplink and downlink data packets to save space. We propose a semi-predictive framework to eliminate the effect of the coupling characteristics on the solution of optimization problem (15). Based on this framework, we remodel this optimization problem to an MDP problem. Note that the semi-predictive framework we proposed is suitable for any value of the uplink and downlink propagation delay. For the generality, we use $d_{\mathrm{up}}=d_{\mathrm{down}}=1$ as an example to illustrate the scheduling strategy design process. In the actual applications with different propagation delay, we only need to modify the value of $d_{\mathrm{up}}$, $d_{\mathrm{down}}$ and adjust some parameters in the following modeling step to meet specific design requirements.

### 3.1. The Packet Outdate Problem

**Figure 2:**
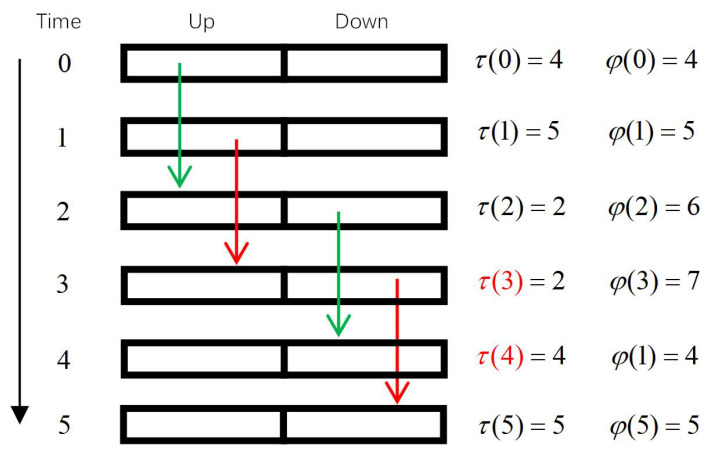
Analysis of Packet Outdated Phenomenon.

Section 2 introduced the control mechanism of a single-loop CPS. Through the above analysis, it is easy to see that state update packets and control command packets have strong coupling characteristic for single-step control methods. Actually, such a coupling characteristic exists in any closed-loop control scenario as long as there exists propagation delay. This characteristic will cause some successfully delivered packets to become outdated. As shown in Figure 2, the green and red arrows represent state update packets ${\mathrm{up}}_{1}$ (left green arrow), ${\mathrm{up}}_{2}$ (left red arrow) and the control command packets ${\mathrm{down}}_{1}$ (right green arrow), ${\mathrm{down}}_{2}$ (right red arrow), respectively. The command ${\mathrm{down}}_{1}$ is generated by the controller using ${\mathrm{up}}_{1}$, while ${\mathrm{down}}_{2}$ is generated by the controller using ${\mathrm{up}}_{2}$. During the period from the slot ${\mathrm{up}}_{2}$ sent to the slot ${\mathrm{down}}_{2}$ executed, if ${\mathrm{down}}_{1}$ is executed successfully, both ${\mathrm{up}}_{2}$ and ${\mathrm{down}}_{2}$ become invalid. In time slot 4, ${\mathrm{down}}_{1}$ is executed; the result is that the real state of the plant was returned to a value around 0. This process causes an interruption in the state estimation process which means the estimation updated by ${\mathrm{up}}_{2}$ is no longer accurate, so ${\mathrm{up}}_{2}$ is outdated. Since ${\mathrm{up}}_{2}$ is outdated, the control command ${\mathrm{down}}_{2}$ which was generated from it is also outdated. This is the main effect of the coupling characteristic and we named it the packet outdate problem.

As we can see, this problem is mainly caused by the discontinuity in the dynamic process of the plant. The discontinuity only occurs when a downlink control command is executed, which means the uplink state update packet will not cause this problem. When this happens, the outdated uplink and downlink data packets require different processing methods. For an outdated downlink packet, it only needs to be discarded. However, for an outdated uplink packet, we have to backtrack the state estimation before this outdated packet is used. We show the evolution of the state estimation age and state control age in Figure 2. It can be seen that the state estimation age has been backtracked by changing from τ(3)=2 to τ(4)=4. The state control age will not be updated like this.

### 3.2. Main Idea of the Semi-Predictive Framework

In the previous subsection, we explained that the packet outdate problem has an impact on the update of the state estimation age, but this problem does not affect the update of the state control age. Therefore, when we try to construct a theoretical analysis framework, as long as the state control age is correct, the final analysis result can be guaranteed to be correct. In other words, the state estimation age of some time slots is allowed to deviate from the actual physical process. As long as it can be ensured that the state estimation age is accurate when the downlink data packet arrives at the actuator, the correct theoretical analysis can be guaranteed. It can be seen that it is possible to skip the state estimation age backtracking process in the theoretical analysis by using this feature. This is the main idea of the semi-predictive framework.

In the normal communication process, the decoding result of a data packet can only be determined after it arrives at the destination. For an uplink data packet, only after it arrives at the controller can it be known whether the data packet can be successfully decoded, while for a downlink packet, only after it arrives at the actuator can it be known whether the data packet can be successfully decoded. However, under the semi-predictive framework, we assume that the transmission result of a downlink packet is known as soon as the downlink packet is sent. Note that we do not predict the result of an uplink packet. This is because the execution of the downlink command is the root cause of the packet outdated problem.

Take the case of Figure 2 as an example again; if we can foresee that the downlink control command packet ${\mathrm{down}}_{1}$ can be successfully decoded and is not outdated, then during the period from its sending to its arrival, any packets sent or arrived can be directly discarded since they will be outdated by ${\mathrm{down}}_{1}$. Through this process, the impact of the packet outdated problem is eliminated and state estimation age backtracking is avoided.

While the update process of the state estimation age under the semi-predictive framework is different from the actual physical process, the scheduling strategy obtained based on this framework can still be directly applied to an actual physical process. In the actual physical process, if a downlink data packet arrives at the actuator successfully and is not outdated, then the uplink and downlink transmissions scheduled during its transmission must be outdated. In other words, no matter what scheduling decision the controller made, those packets sent during this period will be outdated. In other words, those scheduling decisions can be arbitrary since they do not affect the final result. Assuming that the downlink control command packet ${\mathrm{down}}_{1}$ in Figure 2 can be successfully decoded and not outdated, we will explain both age update processes under the semi-predictive framework and the actual physical process in detail.

(1) Semi-Predictive Framework: If ${\mathrm{down}}_{1}$ can be successfully decoded and not outdated, then the controller knows that it does not matter whether it chooses uplink or downlink during the transmission of ${\mathrm{down}}_{1}$ because those scheduled packets will be outdated anyway. Under these circumstance, a reasonable scheduling strategy is to regularly schedule one of the uplink and downlink transmissions during this period to consume time.

(2) Non-Predictive Framework (Actual Physical Process): In the actual physical process, during the transmission of ${\mathrm{down}}_{1}$, the controller continues to schedule uplink or downlink transmissions according to a certain strategy. However, when ${\mathrm{down}}_{1}$ is received and decoded successfully, the previous scheduled transmissions of the controller are all outdated. So in the end, the scheduled transmissions during this period only consume time and have no practical effect.

It can be observed that, under the semi-predictive framework and the actual non-predictive scheduling, the single-loop CPS transmission results are uniform; that is, it is accurate to use the semi-predictive framework in the theoretical design and directly apply the results to the real applications. This subsection qualitatively analyzes the unity of the semi-predictive framework and the actual physical process. In the next subsection, we will quantitatively illustrate how this framework corresponds to actual physical processes through MDP modeling.

### 3.3. MDP Modeling of the Semi-Predictive Framework

Based on the semi-predictive framework, we model the single-loop CPS with uplink and downlink propagation delay as an MDP process with the following four elements:

(1) State Space: The state space of this MDP is

(16) $$S≜\{a^{\prime }\left(-d_{\max}+1\right),\ldots ,a^{\prime }\left(-1\right),a^{\prime }\left(0\right),D\left(0\right),\tau \left(0\right),\varphi \left(0\right)\}$$

where $d_{\max}=\max\left\{d_{\mathrm{up}},d_{\mathrm{down}}\right\}$, $D\left(n\right)\in \left\{0,1,\cdot \cdot \cdot ,d_{\mathrm{down}}+1\right\}$. a(n) represents the scheduling decision made in the time slot *n*. D(n) represents the time interval between the time slot when the latest valid downlink command packet (successfully transmitted and not outdated) in the time slot *n* was generated and the current time slot *n*. τ(n) and φ(n) represent the state estimation age and the state control age at the time slot *n*, respectively. The time slot *n* is based on the current time slot: The time slot for which scheduling decisions are being made. Taking $a^{\prime }\left(-1\right)$ as an example: It represents the transmission action taken in the previous time slot of the current time slot. We set both the uplink and downlink propagation delay to be 1 for illustration in the rest of this paper, so the corresponding state space is: $S≜\{a^{\prime }\left(0\right),D\left(0\right),\tau \left(0\right),\varphi \left(0\right)\}$. In the subsequent sections of this paper, the state space is abbreviated as $S≜\{a^{\prime },D,\tau ,\varphi \}$ to save space.

(2) Action Space: The action space is $A≜\left\{0,1\right\}$. This action space corresponds to the scheduling action $a_{k}$. If the controller schedules uplink transmission in the slot *k*, $a_{k}\;=\;1$. If the controller schedules downlink transmission in the slot *k*, $a_{k}\;=\;2$.

(3) State Transition Probability Matrix: The transition matrix is $P(s^{\prime }|s,a)$. The state transition probability is the probability that the next state is $s^{\prime }$ by taking action *a* in the current state *s*. The transition probability is determined by the channel code error rate. According to the different parameter pairs: $\left(a^{\prime },D\right)$ in the state S, the state transition matrix can be divided into five parts: $\left(a^{\prime },D\right)=\left[\left(1,1\right),\left(1,2\right),\left(2,0\right),\left(2,1\right),\left(2,2\right)\right]$. The complete construction rules are given in Appendix A.

(4) Cost Function: It can be seen from (4) and (11) that the cost function in a specific state is independent of the action. The cost function can be expressed as a function of the state control age $\varphi_{k}$:

(17) $$C\left(s,a\right)=Q_{k}\left(s\right)=f\left(\varphi_{k}\right)\;$$

In the MDP modeling of the semi-predictive framework, the core parameter is D(n). We limit its maximum value to $d_{\mathrm{down}}+1$ because we only need to track the downlink transmissions in the past $d_{\mathrm{down}}$ time slots to ensure that we do not miss any possible packet outdated problems. Besides, such process can help to reduce the scale of the state space. The update rule of D(n) is as follows:

(18) $$D_{k+1}=\left\{\begin{array}{cc} 0 & \mathrm{if}\;(a_{k}\;=\;2)\&(\delta_{k}\;=\;1)\;\\ \max(d_{\mathrm{down}}+1,D_{k}+1) & \mathrm{otherwise} \end{array}\right.$$

This updated process reflects the main idea of the semi-predictive framework and guarantees that it will not cause any differences between the state control ages of the theoretical analysis and the actual physical processes. In the next section, we will use the semi-predictive framework to design the optimal scheduling strategy.

## 4. Online and Offline Scheduling Strategies

In this section, we first give the sufficient condition for the existence of the optimal scheduling strategy. Then we use the relative value iteration algorithm to obtain the lookup table-based optimal offline strategy. Aiming at reducing the space complexity of the algorithm and saving space for storing the optimal offline strategy, we further propose a neural network-based suboptimal online strategy. For different uplink and downlink propagation delay, the acquisition process of both strategies is universal, which means that the semi-predictive framework has high practical application value.

### 4.1. Sufficient Conditions for the Strategies’ Existence

**Theorem 1.**  *(Sufficient conditions for the stability of multi-loop half-duplex CPS with fixed uplink and downlink propagation delay.) Assuming there are K single-loop CPS, all of them share the same controller and form up a multi-loop CPS. If the controller can only schedule L uplink transmissions or L downlink transmissions in each time slot, then for each single-loop CPS i, if the code error probability of its corresponding uplink and downlink channels satisfies*

(19) $$\max\left\{p_{i,up},p_{i,down}\right\}<{\left(\frac{1}{{\left(A_{i}\right)}^{2}}\right)}^{\left\lceil K/L\right\rceil },i\in \left\{1,2,\ldots ,K\right\}$$

*then there must exist a stationary deterministic scheduling strategy that can stabilize the multi-loop CPS. This stability remains as long as the uplink and downlink propagation delay are fixed, but the long term control performance metrics converge to a larger value with the increase of the propagation delay. When*K=1, L=1*the above multi-loop CPS is just a single-loop CPS.The proof is given in Appendix B*.

The essence of this sufficient condition is to link the instability of the control system with the reliability of the communication system. When the reliability of the communication system is higher than the instability of the control system, an optimal scheduling strategy can be found for the communication system to meet the needs of the control system. This condition can effectively guide the design of single- and multi-loop CPS.

### 4.2. Lookup Table-Based Optimal Offline Strategy

**Figure 3:**
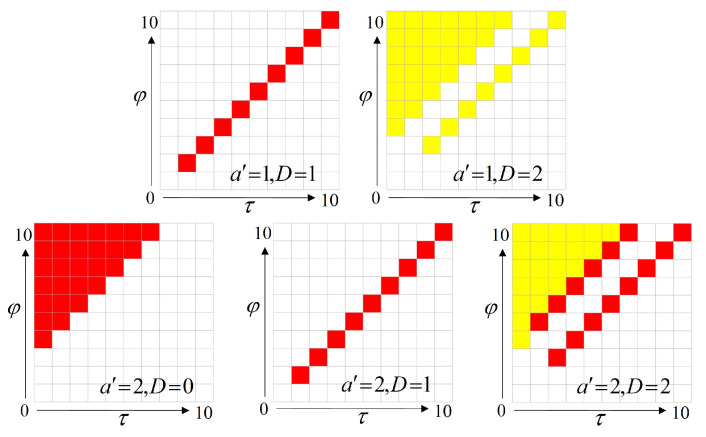
Optimal Off-line Policy with N=10. Red squares represent action a=1; yellow squares represent action a=2.

Since there is no theoretical upper limitation for the state estimation age and the state control age, the scale of the MDP state space is infinite, so it must be truncated before solving. We select N=max{τ,φ} as the truncation condition, and use the relative value iteration algorithm to solve the MDP problem. When the value of *N* is appropriate, this truncation will have no effect on the control performance. Such a suitable *N* can be obtained by conducting Monte Carlo experiments. In this section, we take N=10 as an example to show the resulting scheduling strategy in Figure 3.

In Figure 3, those red squares represent that the controller schedules uplink transmission in the corresponding state, and the yellow squares represent that the controller schedules downlink transmission in the corresponding state. As shown in Figure 3a,c,d, if D={0,1}, no matter which transmission is scheduled, the related packet will be outdated. So under this circumstance, the scheduling strategy can choose any action arbitrarily. Since we chose the relative value iterative algorithm to solve the MDP problem, the strategy we obtained chooses to use uplink transmission to fill these unnecessary transmissions. Note that this part corresponds to the description of Section 3 part C. We take ${\mathrm{down}}_{1}$ as an example again: In the actual physical process, it is not known that the next two transmissions are unnecessary transmissions after ${\mathrm{down}}_{1}$ is sent. The controller does not know that D={0,1}. Instead, it thinks that *D* is still equal to 2 at those time slots. Therefore, the controller continues to schedule according to the scheduling strategy. However, ${\mathrm{down}}_{1}$ makes those two packets outdated when it is executed, while for those states whose D=2, the controller can make a scheduling decision with the right state information. The entire process makes sure that the actual process is consistent with the theoretical process.

After obtaining this scheduling strategy, it is stored as a lookup table by the controller and does not require any extra calculation ability from the controller, so we call it an offline strategy. However, since the iterative algorithm is a model-based algorithm, as *N* gradually increases, the scale of the state space $N_{S}=2\cdot 3\cdot N\cdot N=6N^{2}$ in the MDP modeling increases exponentially. This leads to a sharp increase in the space complexity of the solving process and the lookup table could be too large to be stored. In order to solve these problems, we propose an improved scheme based on neural network in the next subsection.

### 4.3. Neural Network-Based Suboptimal Online Strategy

In Section 3, we remodeled the optimization problem to an MDP problem, and solved it to obtain the optimal offline strategy in the previous subsection. The optimal offline scheduling strategy based on the lookup table has two obvious shortcomings: The size of the lookup table increases linearly as the total number of states in the state space increases and the space complexity required in the calculation process increases exponentially as the total number of states increases. When the optimal offline strategy is actually deployed, there is no guarantee that the central controller has enough storage space to store the entire lookup table. It may even be impossible to perform calculations because the state space is too large. Therefore, here we design a new suboptimal online scheduling strategy based on neural network. The idea of this strategy is to replace the lookup table in the previous strategy with a neural network to save storage space. Neural network is a very ideal approximation function of lookup table, theoretically it can be approximated without error. That means in the theory of reinforcement learning, this strategy can achieve the performance of the optimal strategy. We will show that the performance of this suboptimal online strategy is very close to the performance of the optimal offline strategy in the next section.

In order to obtain this neural network, we use a the model-free algorithm called Deep Q Network (DQN). The algorithm continuously learns the hidden laws of the MDP problem by interacting with the environment and continuously trains the neural network to obtain better performance. We show the detailed process of the algorithm in Algorithm 1.
**Algorithm 1:** Deep Q Network Algorithm.
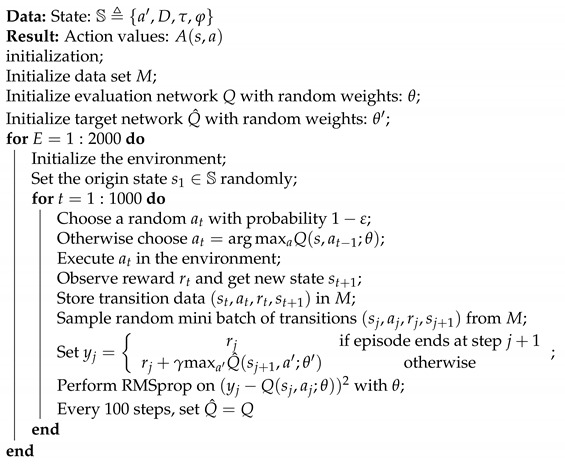


**Figure 4:**
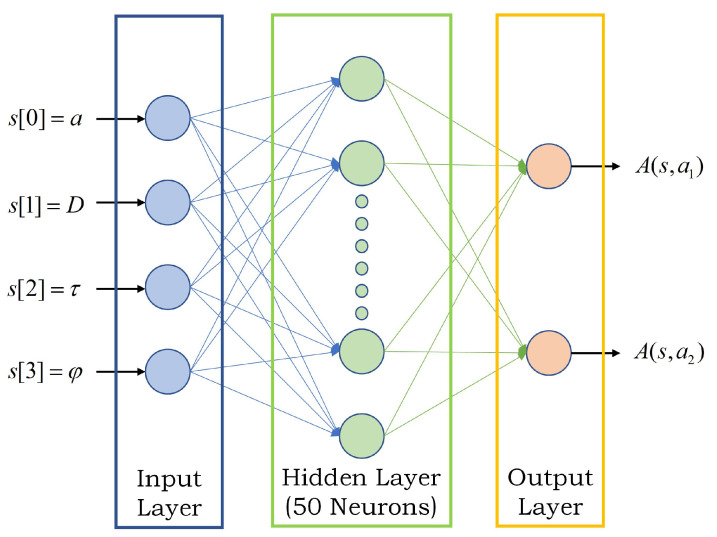
Neural Network Structure.

The structure of the neural network we obtained is shown in Figure 4: Four neurons in the input layer, fifty neurons in the hidden layer, and two neurons in the output layer. This neural network-based scheduling strategy is an online strategy which means that, in order to use this strategy, the current state *s* must be input to the neural network first. Then the controller needs to run real-time calculations to obtain the action values A(s,a) for taking different actions in the current state. The action value represents how much reward can be obtained by taking the action, so the scheduling strategy is to select the action with the largest A(s,a) among all actions.

DQN is a relatively mature reinforcement learning algorithm, so we only give the parameter settings of this algorithm and briefly introduce its training process. We run E=2000 episodes, and each episode contains 1000 steps. In each step, this algorithm executes the greedy strategy with a probability of ε=0.7, and the random strategy with a probability of 1−ε=0.3. After each step, one state transition datum is stored in the data set. The scale of this data set is M=2048, and it is updated in a loop covering manner. A new episode is automatically initialized every 1000 steps. In the meantime, the training process is performed every T=256 steps, the algorithm selects B=512 data from the data set for training. The optimizer we used is the Root Mean Square prop optimizer (RMSprop).

With the help of the DQN algorithm, we can obtain the neural network-based suboptimal online strategy. The controller only needs to store the node value of this network, and then calculates the action value in real time according to the current state in each time slot. In other words, this strategy saves a lot of storage space by consuming a small amount of computing ability of the controller. Such an advantage makes this strategy very meaningful in practical applications.

## 5. Numerical Simulation

In this section, we run the numerical simulation on those strategies we proposed and some existing strategies. We illustrate the advantages of the proposed strategies through comparison. First we introduce two benchmark strategies. The first is the switch scheduling strategy, that is, alternate uplink and downlink transmissions between each time slot; the second is the insist scheduling strategy, that is, continuous scheduling of uplink or downlink transmissions until success, then the transmission is exchanged.

The parameter settings in the numerical simulation are as follows: The state transition coefficient is A=[1.1,1.3], the code error rates of the uplink and downlink channels are $p_{s}=p_{c}=\left[0.1,0.2\right]$, the specific values are marked on the curve obtained from the simulation. The initial state of the plant is $X_{0}=1$. The noise distribution is $N(\bar{z}=0,$R=1). The command control coefficient is B=−A. The initial state control variable is $s_{0}=\left(a_{0},D_{0},\tau_{0},\varphi_{0}\right)=\left(1,1,2,2\right)$. The corresponding initial scheduling action is $a_{0}=1$. The initial state of the controller estimation is ${\tilde{X}}_{o}=1$. The range of truncated state space is N=max{τ,φ}=20. The plant noise follows normal distribution $N(\bar{z}=0,R=1)$. Each strategy runs 500 episodes with 10,000 time slots each episode. The final long-term average plant state MSE is the average of the results of 500 episodes.

**Figure 5:**
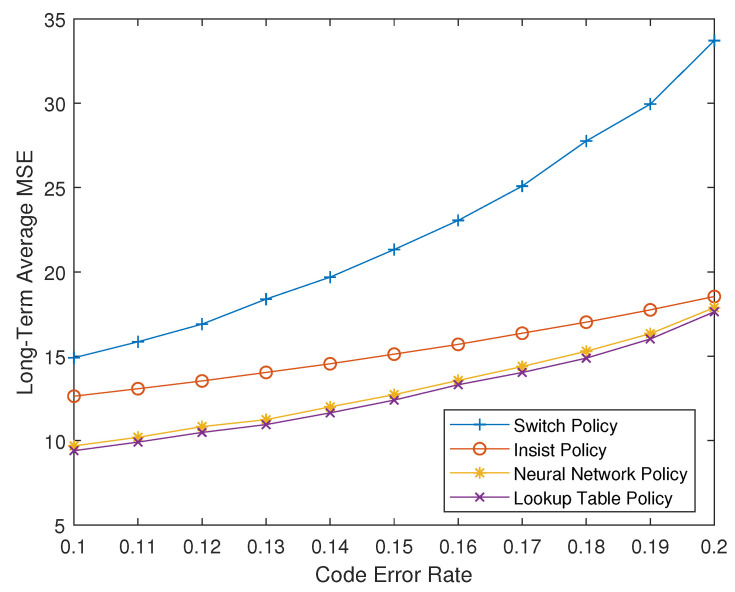
Long-term average plant state MSE of four policies with A=1.3 and $p_{s}=p_{c}=\left[0.1,0.2\right]$.

Figure 5 show the long-term average MSE of four strategies with A=1.3 and $p_{s}=p_{c}=\left[0.1,0.2\right]$. It can be seen that the MDP strategy, that is, the optimal offline strategy, has the best performance among all strategies, which also is the best performance that all possible scheduling strategies can achieve. While the performance of the neural network-based online strategy has slightly decreased, it is still significantly ahead of the existing strategies, and the performance gap between the optimal offline strategy and the suboptimal online strategy is very small. This gap can be eliminated in theory, but due to the limitations of deep reinforcement learning technology, it is currently difficult to fully achieve the optimal performance. It is relatively simple to obtain a suboptimal strategy with very close performance.

**Figure 6:**
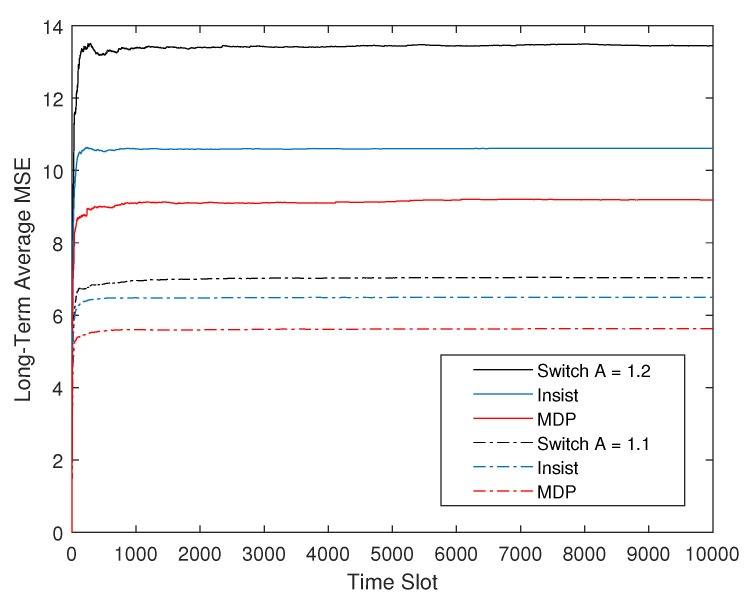
Long-term average plant state MSE of three policies with $p_{s}=p_{c}=0.2$ and A={1.1,1.2}.

Figure 6 show the performance comparison between the optimal offline strategy and the two existing strategies under different state transition coefficient *A*. The suboptimal online strategy is not shown because it has been explained that the suboptimal strategy can theoretically approach the optimal. The state transition coefficient and the channel code error rates both reflect the instability of the control system and the reliability of the communication system in Equation (19). Combined with Figure 5, it can be seen that their influence on CPS is the same. A larger state transition coefficient or a higher channel code error rate lead to an increase in the long-term average plant state MSE, and when they exceed a certain limit and no longer satisfy Equation (19), the long-term average MSE of the CPS no longer converges, which means the single-loop CPS is unstable.

## 6. Conclusions

We proposed the semi-predictive framework to design scheduling strategies for single-loop CPS with uplink and downlink propagation delay. This framework can obtain the optimal offline strategy which is the upper bound on the performance among all strategies and a suboptimal online strategy with more practical application value. By adjusting the parameters, the semi-predictive framework can meet the need of any practical applications. We introduced the complete process of designing scheduling strategies under this framework by taking a specific situation as an example. The numerical simulation proved that the obtained strategies can effectively improve the performance of the existing strategies.

## Figures and Tables

**Figure A1:**
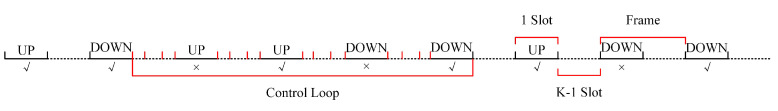
The round-robin insist scheduling strategy.

## Data Availability

Not applicable.

## Appendix A. Construction Rules of the State Transition Probability Matrix

Here we give the complete construction rules of the state transition matrix. Firstly, we give all the possible new states after a state transition as follows:

(A1) $$s_{1}^{\prime }=\left(1,2,\tau +1,\varphi +1\right)$$

(A2) $$s_{2}^{\prime }=\left(2,2,\tau +1,\varphi +1\right)$$

(A3) $$s_{3}^{\prime }=\left(2,0,\tau +1,\varphi +1\right)$$

(A4) $$s_{4}^{\prime }=\left(1,2,1,\varphi +1\right)$$

(A5) $$s_{5}^{\prime }=\left(2,2,1,\varphi +1\right)$$

(A6) $$s_{6}^{\prime }=\left(2,0,1,\varphi +1\right)$$

(A7) $$s_{7}^{\prime }=\left(1,1,\tau +1,\tau +1\right)$$

(A8) $$s_{8}^{\prime }=\left(2,1,\tau +1,\tau +1\right)$$

Secondly, we use *R* and $R^{\prime }$ to mark the transmission results. *R* represents the result of the downlink transmission scheduled in the next time slot. $R^{\prime }$ represents the result of the uplink transmission arrived in the next time slot. Note that *R* is known by prediction while $R^{\prime }$ is known by normal communication process. These abbreviations can help to simplify the expression of the rules.

We will give the construction rules in the form of $P[s^{\prime }|s,c]=p$ which means that when the condition *c* is satisfied, the previous state *s* transfers to the new state $s^{\prime }$ with a probability of *p*.

*When s=(1,1,τ,φ):*

(A9) $$\begin{array}{c} P[{s^{\prime }}_{1}|s,a=1]=1\\ P[{s^{\prime }}_{2}|s,a=2]=1 \end{array}$$

*When s=(2,0,τ,φ):*

(A10) $$\begin{array}{c} P[{s^{\prime }}_{7}|s,a=1]=1\\ P[{s^{\prime }}_{8}|s,a=2]=1 \end{array}$$

*When s=(2,1,τ,φ):*

(A11) $$\begin{array}{c} P[{s^{\prime }}_{1}|s,a=1]=1\\ P[{s^{\prime }}_{2}|s,a=2]=1 \end{array}$$

*When s=(2,2,τ,φ):*

(A12) $$\begin{array}{c} P[{s^{\prime }}_{1}|s,a=1]=1\\ P\left[{s^{\prime }}_{2}|s,a=2,R=0\right]=p_{c}\\ P\left[{s^{\prime }}_{2}|s,a=2,R=1,\tau =\varphi \right]=p_{s}\cdot \left(1-p_{c}\right)\\ P\left[{s^{\prime }}_{3}|s,a=2,R=1,\tau \neq \varphi \right]=p_{s}\cdot \left(1-p_{c}\right) \end{array}$$

*When s=(1,2,τ,φ):*

(A13) $$\begin{array}{c} P\left[{s^{\prime }}_{1}|s,a=1\right]=p_{s}\\ P\left[{s^{\prime }}_{2}|s,a=2,R^{\prime }=0,R=0\right]=p_{s}\cdot p_{c}\\ P\left[{s^{\prime }}_{2}|s,a=2,R^{\prime }=0,R=1,\tau =\varphi \right]=p_{s}\cdot \left(1-p_{c}\right)\\ P\left[{s^{\prime }}_{3}|s,a=2,R^{\prime }=0,R=1,\tau \neq \varphi \right]=p_{s}\cdot \left(1-p_{c}\right)\\ P\left[{s^{\prime }}_{4}|s,a=1,R^{\prime }=1\right]=1-p_{s}\\ P\left[{s^{\prime }}_{5}|s,a=2,R^{\prime }=1,R=0\right]=\left(1-p_{s}\right)\cdot p_{c}\\ P\left[{s^{\prime }}_{6}|s,a=2,R^{\prime }=1,R=1\right]=\left(1-p_{s}\right)\cdot \left(1-p_{c}\right) \end{array}$$

## Appendix B. Proof of Theorem 1

### Appendix B.1. Scheduling 1 Subsystem per Time Slot without Delay

To prove sufficient conditions, we only need to prove that there exists a stationary deterministic strategy that can make multi-loop CPS stable. Here we prove that the round-robin insist scheduling strategy can keep the system stable. We first prove the case of L=1. Round-robin means that in every *K* time slots, the controller schedules each subsystem once in turn, and the scheduling sequence is fixed from i=1 to i=K. Insist refers to when scheduling each subsystem, continuously scheduling uplink or downlink transmission until it succeeds, then switch to another transmission. Therefore, the actions of a single subsystem under the round-robin insist scheduling strategy can be given in the form of the following time axis:

The time axis between two consecutive successful downlink scheduling is recorded as a control loop. It can be seen that the AoI evolution process of each control loop of one subsystem is:

(1) The initial estimation age is equal to the control age: $n^{\prime }K$
(2) The current subsystem waits for the completion of the scheduling of other subsystems, that is, silence (k−1) time slots, and then schedules the uplink transmission when it is scheduled again. If the uplink transmission fails, the subsystem waits another (*k* − 1) time slots and tries again until the uplink transmission is successful. This step takes mK time slots. At the end of this step, the estimated age is 0, and the control age is $(n^{\prime }+m)K$;
(3) After the current subsystem silences for (K−1) time slots, it switches to schedule downlink transmission continuously until it succeeds. This step takes nK time slots. At the end of this step, the estimated age is equal to the control age: nK. Then it finishes a close control loop.

Note that the time slots included in a complete control loop are the time slots marked in red on the coordinate axis in Figure A1, that is, the control age ranges from $n^{\prime }K$ to $(n^{\prime }+m+n)K$. Each control loop has repeatability, so we only need to prove that the long-term average cost within the range of one control loop converges.

According to the channel error probability, the *M* uplink transmissions and *N* downlink transmissions in each control loop can be modeled as a geometric distribution with the probability of success being $\left(1-p_{s}\right)$ and $\left(1-p_{c}\right)$ respectively. *M* and *N* are different in each control loop, $N^{\prime }$ Represents the number of downlink transmissions in the previous loop of the current control loop. $\left(n^{\prime },m,n\right)$ are their specific observations. $C_{i}$ and $T_{i}$ represent the total cost and total time of the *i*-th control loop of the current subsystem respectively:

(A14) $$C_{i}=\sum_{q=0}^{(m+n)K-1}f(n^{\prime }K+q)=\sum_{q=1}^{(m+n)K}f(n^{\prime }K+q-1)$$

(A15) $$T_{i}=\left(m+n\right)K$$

where $f\left(\varphi \right)=\sum_{i=1}^{\varphi }{\left(A^{2}\right)}^{i-1}$. Next, we can express the long-term average cost as:

(A16) $$J=\lim_{t\to \infty }\frac{C_{1}+C_{2}+\cdot \cdot \cdot +C_{t}}{T_{1}+T_{2}+\cdot \cdot \cdot +T_{t}}=\frac{E[C]}{E[T]}$$

(A17) $$E\left[C\right]=\sum_{n^{\prime }}^{\infty }\sum_{m}^{\infty }\sum_{n}^{\infty }\left(\begin{array}{c} E[C|N^{\prime }=n^{\prime },M=m,N=n]\\ \;\cdot P[N^{\prime }=n^{\prime },M=m,N=n] \end{array}\right)$$

(A18) $$E\left[L\right]=\sum_{n^{\prime }}^{\infty }\sum_{m}^{\infty }\sum_{n}^{\infty }\left(\left(m+n\right)\cdot K\cdot P\left[N^{\prime }=n^{\prime },M=m,N=n\right]\right)$$

It can be seen that if E[C] is bounded, then *J* is bounded. According to the definition of $C_{i}$ and the three geometrical distributions $\left(N^{\prime },M,N\right)$, which are independent of each other, we have:

(A19) $$\begin{array}{c} E\left[C|N^{\prime }=n^{\prime },M=m,N=n\right]=\sum_{q=0}^{(m+n)K-1}f(n^{\prime }K+q)=\sum_{q=1}^{(m+n)K}f(n^{\prime }K+q-1) \end{array}$$

(A20) $$\begin{array}{c} \begin{array}{c} P[N^{\prime }=n^{\prime },M\;=\;m,N\;=\;n]\\ =P\left[N^{\prime }=n^{\prime }\right]\cdot P\left[M=m\right]\cdot P\left[N=n\right]\\ =\left(1-p_{c}\right){p_{c}}^{n^{\prime }-1}\left(1-p_{s}\right){p_{s}}^{m-1}\left(1-p_{c}\right){p_{c}}^{n-1} \end{array} \end{array}$$

Choose $p_{\max}=\max\left\{p_{s},p_{c}\right\}$, we can derive that:

(A21) $$E\left[C\right]⩽\alpha_{1}\cdot \sum_{n^{\prime }}^{\infty }\sum_{m}^{\infty }\sum_{n}^{\infty }\left(\begin{array}{c} \sum_{q=1}^{(m+n)K}f(n^{\prime }K+q-1)\cdot {p_{\max}}^{n^{\prime }+m+n} \end{array}\right)$$

where $\alpha_{1}=\left(1-p_{c}\right){p_{c}}^{-1}\left(1-p_{s}\right){p_{s}}^{-1}\left(1-p_{c}\right){p_{c}}^{-1}$. Since f(·) is a strictly increasing function and $\left(n^{\prime },m,n\right)$ are all greater than 0, we can derive that:

(A22) $$E\left[C\right]<\alpha_{2}\cdot \sum_{n^{\prime }}^{\infty }\sum_{m}^{\infty }\sum_{n}^{\infty }\left(\begin{array}{c} \left(n^{\prime }\;+\;m+n\right)\;\cdot f\left(n^{\prime }K+mK+nK\right)\;\cdot {p_{\max}}^{n^{\prime }+m+n} \end{array}\right)$$

where $\alpha_{2}=K\left(1-p_{c}\right){p_{c}}^{-1}\left(1-p_{s}\right){p_{s}}^{-1}\left(1-p_{c}\right){p_{c}}^{-1}$. We abbreviate $n^{\prime }+m+n$ as *i*, that is, $i=n^{\prime }+m+n$. Considering i⩾3, and when $i=n^{\prime }+m+n$ is a fixed value, the possible combinations of $(n^{\prime },m,n)⩾1$ satisfy the mathematical relationship of $\sum_{n^{\prime }}^{}\sum_{m}^{}\sum_{n}^{}\left(1\right)<{\left(n^{\prime }+m+n\right)}^{3}$, namely:

(A23) $$\sum_{n^{\prime }}^{}\sum_{m}^{}\sum_{n}^{}\left(n^{\prime }+m+n\right)<{\left(n^{\prime }+m+n\right)}^{3}\cdot \left(n^{\prime }+m+n\right)$$

(A24) $$\sum_{n^{\prime }}^{}\sum_{m}^{}\sum_{n}^{}\left(i\right)<{\left(i\right)}^{4}$$

We can derive that:

(A25) $$E\left[C\right]<\alpha_{2}\cdot \sum_{i}^{\infty }\left(i^{4}\cdot f\left(iK\right)\cdot {p_{\max}}^{i}\right)$$

Since there are always exist $p>p_{\max}$ and n<∞, satisfying $i^{4}{p_{\max}}^{i}<p^{i},\;\forall i>n$. So we have:

(A26) $$\sum_{i}^{\infty }\left(i^{4}\cdot f\left(iK\right)\cdot {p_{\max}}^{i}\right)<\sum_{i}^{\infty }\left(f\left(iK\right)\cdot p^{i}\right)$$

So if $\sum_{i}^{\infty }\left(f\left(iK\right)\cdot p^{i}\right)<\infty$, then $\sum_{i}^{\infty }\left(i^{4}\cdot f\left(iK\right)\cdot {p_{\max}}^{i}\right)<\infty$. Now seeking the conditions for the stability of the multi-loop CPS subsystem is transformed into seeking the conditions for the establishment of $\sum_{i}^{\infty }\left(f\left(iK\right)\cdot p^{i}\right)<\infty$. For f(iK), we have:

(A27) $$\begin{array}{c} f\left(iK\right)=\sum_{q=1}^{iK}{\left(A^{2}\right)}^{q-1}=1+A^{2}+A^{4}+\cdot \cdot \cdot +A^{2(iK-1)}=\frac{1-{\left(A^{2}\right)}^{iK}}{1-A^{2}} \end{array}$$

For $\sum_{i}^{\infty }\left(f\left(iK\right)\cdot p^{i}\right)$, we have:

(A28) $$\begin{array}{c} \sum_{i}^{\infty }\left(f\left(iK\right)\cdot p^{i}\right)\;=\;\sum_{i}^{\infty }\left(\frac{1-{\left(A^{2}\right)}^{iK}}{1-A^{2}}\cdot p^{i}\right)=\frac{1}{1-A^{2}}\sum_{i}^{\infty }\left(\left(1-{\left(A^{2}\right)}^{iK}\right)\cdot p^{i}\right)\\ =\frac{1}{1-A^{2}}\left(\sum_{i}^{\infty }\left(p^{i}-{\left(A^{2}\right)}^{iK}p^{i}\right)\right)=\frac{1}{1-A^{2}}\left(\sum_{i}^{\infty }\left(p^{i}\right)-\sum_{i}^{\infty }\left({\left(A^{2}\right)}^{iK}p^{i}\right)\right) \end{array}$$

So in order to ensure that $\frac{1}{1-A^{2}}\left(\sum_{i}^{\infty }\left(p^{i}\right)-\sum_{i}^{\infty }\left({\left(A^{2}\right)}^{iK}p^{i}\right)\right)<\infty$ is satisfied, it is obvious that p<1 and $A^{2K}p<1$ must stand, that is, $p<{\left(\frac{1}{A^{2}}\right)}^{K}$. This completes the proof.

### Appendix B.2. Scheduling L Subsystems per Time Slot without Delay

When each time slot can schedule *L* subsystems, the corresponding strategy can be set to multiple independent round-robin insist scheduling strategies. It can be ensured that the round-robin cycle of each subsystem does not exceed $\left\lceil K/L\right\rceil$, and the follow-up proof is consistent with Appendix B.1.

### Appendix B.3. Scheduling L Subsystems per Time Slot with Delay

For a specific subsystem, we assume that the fixed delay for each transmission is $D_{i}$ frames, which is equivalent to the uplink and downlink scheduling in the control lcfoop must be delayed by $D_{i}K$ time slots for AA reception, so the formula (A19) is modified as follows:

(A29) $$\begin{array}{c} E[C|N^{\prime }=n^{\prime },M=m,N=n]\\ =\sum_{q=0}^{(m+n\;+\;2D)K-1}f(n^{\prime }K+DK+q)\\ =\sum_{q=1}^{(m+n\;+\;2D)K}f(n^{\prime }K+DK+q-1) \end{array}$$

Since $E\left[D\right]=E\left[D_{i}\right]=D$ is a constant which has no effect on the subsequent proof, the proof process is consistent with Appendix B.1.
